# DGCR8-dependent efficient pri-miRNA processing of human pri-miR-9-2

**DOI:** 10.1016/j.jbc.2021.100409

**Published:** 2021-02-10

**Authors:** Masahiro Nogami, Kazumasa Miyamoto, Yoshika Hayakawa-Yano, Atsushi Nakanishi, Masato Yano, Hideyuki Okano

**Affiliations:** 1 Innovative Biology Laboratories, Neuroscience Drug Discovery Unit, Research, Takeda Pharmaceutical Company Limited, Fujisawa, Kanagawa, Japan; 2 Shonan Incubation Laboratories, Takeda Pharmaceutical Company Limited, Fujisawa, Kanagawa, Japan; 3 Drug Safety Research Laboratories, Takeda Pharmaceutical Company Limited, Fujisawa, Kanagawa, Japan; 4 Division of Neurobiology and Anatomy, Graduate School of Medical and Dental Sciences, Niigata University, Niigata, Japan; 5 Regenerative Medicine Unit, Takeda Pharmaceutical Company Limited, Fujisawa, Kanagawa, Japan; 6 Department of Physiology, School of Medicine, Keio University, Tokyo, Japan

**Keywords:** pri-miR-9-2, DGCR8, miRNA, pri-miRNA processing, DiGeorge syndrome, schizophrenia, ribonuclease, neurogenesis, and fluorescence, DGCR8, DiGeorge syndrome critical region gene 8, DRE, DGCR8-responsive RNA element, poly(A), polyadenylation, pri-miRNA, primary transcripts of microRNA, CLIP, UV-cross-linking and immunoprecipitation

## Abstract

Microprocessor complex, including DiGeorge syndrome critical region gene 8 (DGCR8) and DROSHA, recognizes and cleaves primary transcripts of microRNAs (pri-miRNAs) in the maturation of canonical miRNAs. The study of DGCR8 haploinsufficiency reveals that the efficiency of this activity varies for different miRNA species. It is thought that this variation might be associated with the risk of schizophrenia with 22q11 deletion syndrome caused by disruption of the *DGCR8* gene. However, the underlying mechanism for varying action of DGCR8 with each miRNA remains largely unknown. Here, we used *in vivo* monitoring to measure the efficiency of DGCR8-dependent microprocessor activity in cultured cells. We confirmed that this system recapitulates the microprocessor activity of endogenous pri-miRNA with expression of a ratiometric fluorescence reporter. Using this system, we detected mir-9-2 as one of the most efficient targets. We also identified a novel DGCR8-responsive RNA element, which is highly conserved among mammalian species and could be regulated at the epi-transcriptome (RNA modification) level. This unique feature between DGCR8 and pri-miR-9-2 processing may suggest a link to the risk of schizophrenia.

MicroRNAs (miRNAs) are small noncoding RNAs that regulate gene expression through specifically targeting mRNAs for degradation and translation inhibition (1, 2, 3). miRNAs are initially produced as long primary transcripts (pri-miRNAs), which are processed by the microprocessor complex composed of ribonuclease III, Drosha, and the RNA-binding protein DiGeorge syndrome critical region gene 8 (DGCR8) (4, 5, 6, 7, 8, 9, 10). Precursor miRNAs (pre-miRNAs) are exported from the nucleus into the cytoplasm by exportin-5, processed again by Dicer, and then loaded into the RNA-induced silencing complex (11, 12).

Several RNA-binding proteins, including DGCR8, function in pri-miRNA processing and are involved in neuronal development and diseases such as DiGeorge syndrome (1, 13, 14). The human nervous system expresses 70% of known miRNAs, and several brain-specific miRNAs have recently been identified (15). Among these, miR-9 is expressed specifically in neurogenic regions of the brain during neural development and adulthood (16, 17, 18, 19, 20, 21). miR-9 is encoded by three pri-miR-9 genes, pri-miR-9-1, pri-miR-9-2, and pri-miR-9-3, in human and mouse genomes (20). In humans, pri-miR-9 can be transcribed from chromosomes 1 (pri-miR-9-1), 5 (pri-miR-9-2), and 15 (pri-miR-9-3); the mature miRNA sequences generated from all three loci are identical. Only pre-miR-9-2 is expressed in neural stem cells differentiated from human induced pluripotent stem cells (22), and neither miR-9-1 nor miR-9-3 show robust expression in the developing human brain; miR-9-2 expression peaks by postgestation 16 weeks (23). A recent genome-wide association study (24), Schizophrenia Working Group of the Psychiatric Genomics Consortium, showed that the miR-9-2 gene is located near a single nucleotide polymorphism locus associated with schizophrenia with genome-wide significance (24). Moreover, a gene set enrichment analysis using summary statistics from the Psychiatric Genomics Consortium found an enrichment of predicted miR-9 target mRNAs among schizophrenia-associated genes (24). These results suggest that genetic variants in both miR-9 and its targets are associated with an increased risk of schizophrenia.

DiGeorge syndrome is caused by the chromosomal deletion 22q11.2, where the *DGCR8* gene is located, and patients often show cognitive and behavioral impairment (25, 26); in fact, this deletion is one of the well-established risk factors for the development of schizophrenia (27, 28). The mouse model for haploinsufficiency of the *Dgcr8* gene shows abnormal miRNA biogenesis caused by decreased *Dgcr8* gene expression, schizophrenia-like deficits, and decreased neurogenesis in the adult hippocampus (29, 30). Of interest, this model also revealed that only a subset of pri-miRNA genes is upregulated and that a smaller subset of mature miRNAs is downregulated (29). pri-miR-9-2 is one of the most upregulated pri-miRNAs in the adult hippocampus of model mice, supporting the fact that DGCR8 more efficiently processes miR-9-2 than other miRNA species. Considering that miR-9 is a critical miRNA for the differentiation of neural precursor cells (21), the DGCR8–miR-9-2 axis is thought to be involved in neurogenic differentiation and a cause of schizophrenia.

In this study, we address the underlying mechanism of how DGCR8 efficiently processes pri-miR-9-2 to contribute to the generation of miR-9. To analyze this efficiency, we performed a cellular optimized pri-miRNA processing assay using a ratiometric fluorescence reporter, based on a similar system reported previously (31). Using this assay in combination with various types of miRNAs and pri-miR-9 mutant reporters, we provide clear evidence that pri-miR-9-2 has a novel DGCR8-responsive RNA element (DRE) that is well conserved among mammalian species and promotes DGCR8-dependent pri-miRNA processing activity.

## Results

### Fluorescence-based live-cell pri-miR-9-1 processing reporter system

**Figure 1:**
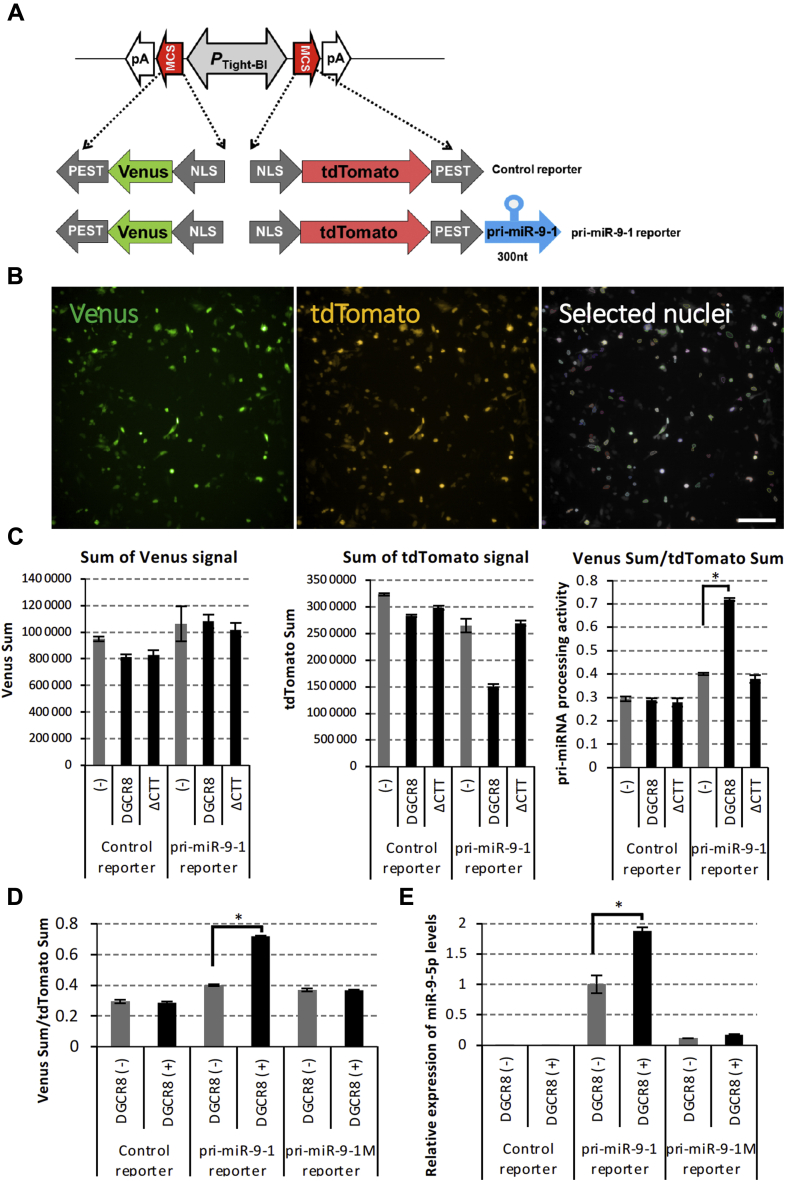
**Live-cell pri-miRNA processing reporter assay.***A*, schematic of fluorescent reporter vector construction. Venus and tdTomato mRNAs were transcribed under the Tet-On-responsive bidirectional promoter (P_Tight-BI_). The 300-nt cDNA coding human pri-miR-9-1 was subcloned into a multicloning site (MCS) in the 3′-UTR of tdTomato mRNA. A microprocessor complex including Drosha and DGCR8 cleaves pri-miRNA to produce pre-miRNA from the 3′-UTR of tdTomato mRNA, which is destabilized because the poly(A) sequence is removed from the 3′-UTR. pA, poly(A) signal sequence. *B*, After transfecting the fluorescent reporter vector into HeLa Tet-On 3G cells, nuclear expression of Venus and tdTomato was observed by Opera Phenix, a high content cell imaging analyzer. The scale bar represents 200 μm. *C*, After transfecting the fluorescent reporter control, the pri-miRNA or pri-miR-9-1 reporter vector was transfected with pcDNA3.1 or the FLAG-DGCR8 expression vector into HeLa Tet-On 3G cells. The sums of the Venus fluorescent signal intensity and tdTomato fluorescent signal intensity in selected nuclei were shown in the graph. The relative sum of the Venus signal intensity to the sum of the tdTomato signal intensity was calculated for each well and relative values are shown. Error bars show the standard deviation (n = 3). *D*, fluorescence pri-miRNA processing reporter assay. Control, pri-miR-9-1, and pri-miR-9-1M were transfected with pcDNA3.1 or FLAG-DGCR8 expression vectors into HeLa Tet-On 3G and fluorescent signals were monitored from each cell. The relative sum of the Venus signal intensity to the sum of the tdTomato signal intensity was calculated in each well and shown. The error bar shows the standard deviation (n = 3). *E*, has-miR-9-5p was quantified by qRT-PCR with total RNA purified from HeLa Tet-On 3G cells transiently transfected with control, pri-miR-9-1, pri-miR-9-1M reporter, and FLAG-DGCR8 expression vectors. The error bar shows the standard deviation (n = 3). The *asterisk* indicates significant change (*t*-test *p* < 0.001); NLS, nuclear localization signal; PEST, a peptide sequence that acts as a signal peptide for protein degradation.

To analyze the DGCR8-dependent pri-miRNA processing activity of pri-miR-9, we performed a live-cell pri-miRNA processing assay using a ratiometric fluorescence reporter, based on a similar system reported previously for examining pri-miRNA processing activities regulated by ectopically expressing DGCR8 and its mutants (31). We further optimized a ratiometric reporter construct based on a plasmid that simultaneously expresses two fluorescent proteins, tdTomato and Venus-fused PEST sequence, a signal peptide for protein degradation, driven by a bidirectional tetracycline-inducible promoter (Fig. 1*A*). In our reporter construct, pri-miRNA sequences were subcloned into the 3′-UTR of the tdTomato expression cassette, whereas the Venus 3′-UTR was intact. This construct expresses tdTomato-encoding mRNA containing pri-miRNA-fused 3′-UTR RNA. Once the Drosha/DGCR8 microprocessor complex cleaves pri-miRNA in the 3′-UTR, the polyadenylation (poly(A)) signal from tdTomato mRNA is removed, causing tdTomato protein expression to decrease because of mRNA and protein degradation. Of importance, the Drosha/DGCR8 microprocessor complex does not affect the Venus fluorescence signal associated with individual cell transcriptional and translational activities. Thus, the tdTomato:Venus ratio should negatively correlate with pri-miRNA processing activity.

In this study, we used the reciprocal Venus:tdTomato ratio as a positive indicator of pri-miRNA processing efficiency, as described previously (31). First, we engineered 300 nucleotides (nt) of human pri-miR-9-1 into our fluorescent reporter. HeLa Tet-On 3G cells transiently transfected with control or pri-miR-9-1 fluorescent reporter showed nuclear expression of Venus and tdTomato because of the presence of an N-terminal nuclear localization signal tag. The Venus and tdTomato signal intensity was monitored, and nuclei with a Venus signal were selected (Fig. 1*B*). Then, Venus signals were calculated relative to the tdTomato signal for each well as an indicator of pri-miRNA processing efficiency (Fig. 1*C*). Ectopically overexpressing N-terminal FLAG-tagged DGCR8 (FLAG-DGCR8) promoted pri-miRNA processing activity in the fluorescence-based live-cell pri-miRNA processing reporter assay, as reported previously (31).

The C-terminal tail of DGCR8 was previously shown to be required for pri-miRNA processing (31, 32, 33), and a mutant with the C-terminal tail deleted did not promote pri-miRNA processing activity, as reported previously. We also constructed a mutant pri-miR-9-1 reporter pri-miR-9-1M, which did not respond to ectopic FLAG-DGCR8 expression because of four point mutations within the pri-miR-9-1 cleavage site (Figs. 1*C* and S1). The fluorescent miRNA processing assay was performed with the pri-miR-9-1M reporter vector. Ectopically expressing FLAG-DGCR8 promoted pri-miRNA processing activity in cells transfected with the pri-miR-9-1 reporter but not in those transfected with the pri-miR-9-1M reporter (Fig. 1*D*). Moreover, the production of miR-9-5p was decreased in cells transfected with the pri-miR-9-1M reporter (Fig. 1*E*). Taken together, these results indicate that our fluorescent reporter system was able to monitor pri-miR-9-1 processing activities in an ectopically expressing DGCR8-dependent manner, as reported previously.

### Pri-miR-9-2 is processed by the canonical microprocessor complex

**Figure 2:**
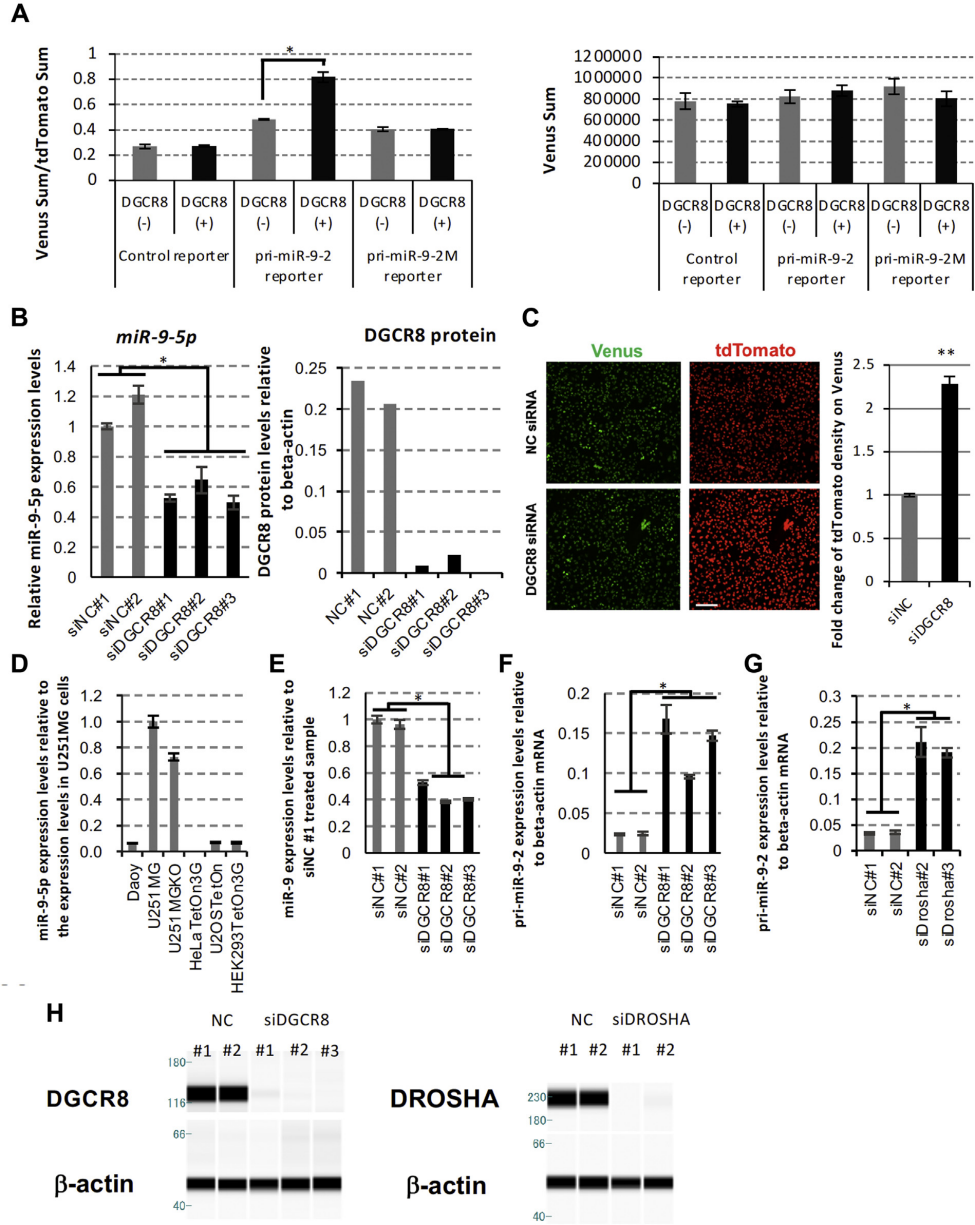
**pri-miR-9-2 is processed by the microprocessor complex.***A*, the HeLa Tet On 3G clone stably expressing the pri-miR-9-2 reporter can monitor the cellular microprocessor complex activity. Venus and tdTomato proteins were induced by doxycycline treatment in a dose-dependent manner. Negative control siRNA (siNC#1) and DGCR8 siRNA (siDGCR8#1) were transfected into the cells, and nuclear expression of Venus and tdTomato was observed by the cell imaging analyzer. *B*, negative control siRNAs (siNC#1 and #2) and DGCR8 siRNAs (siDGCR8#1–#3) were transfected into HeLa Tet On 3G cells with pri-miR-9-2 reporter or control reporter vectors. The amount of endogenous miR-9-5p was quantified with the specific TaqMan qRT-PCR system. miR-9-5p production was suppressed by transfecting DGCR8 siRNAs. DGCR8 proteins were knocked down by DGCR8 siRNAs. *C*, HeLa Tet On 3G cells stably expressing the pri-miR-9-2 reporter were treated with siNC and siDGCR8, and Venus and tdTomato expression was observed by the cell imaging analyzer (*left*). The scale bar represents 500 μm. The fold-change of the tdTomato fluorescence signal intensity is shown in the graph. *D*, hsa-miR-9-5p was quantified by qRT-PCR with total RNA purified from human cell lines Daoy, U251MG, U251MG(KO), HeLa Tet-On 3G,U2OSTteOn, and HEK293TetOn3G. The error bar shows the standard deviation (n = 3). *E*, negative control siRNAs (siNC#1 and #2) and DGCR8 siRNAs (siDGCR8#1, #2, and #3) were transfected into U251MGKO cells, and the amount of endogenous miR-9-5p was quantified. *F* and *G*, the amount of endogenous pri-miR-9-2 was quantified in U251MGKO cells treated with DGCR8 siRNAs (*F*) and Drosha siRNAs (*G*). *H*, negative control siRNAs (siNC#1 and #2), DGCR8 siRNAs (siDGCR8#1, #2, and #3), and Drosha siRNAs (siDrosha#2 and #3) were transfected into U251MGKO cells, and DGCR8 and β-actin protein expression was analyzed with the Wes protein analysis system. The *asterisk* indicates significant change (∗ *p* < 0.001, ∗∗ *p* < 0.01)

To analyze pri-miR-9-2 processing, we similarly constructed the pri-miR-9-2 fluorescence reporter. Ectopically expressing DGCR8 promoted pri-miRNA processing activity in cells expressing pri-miR-9-2 but not in those expressing the pri-miR-9-2M reporter (Fig. 2*A*). Thus, we confirmed that pri-miR-9-2 processing was sensitive to the ectopic expression of both DGCR8 and pri-miR-9-1. Next, DGCR8 siRNAs were transfected into HeLa Tet-On 3G cells to reduce DGCR8 protein expression, and miR-9-5p levels were quantified. DGCR8 siRNAs effectively knocked down DGCR8 protein and reduced miR-9-5p expression levels compared with negative control siRNAs (Fig. 2*B*). DGCR8 siRNA also promoted tdTomato-derived fluorescence signals relative to Venus (Fig. 2*C*). Thus, pri-miR-9-2 reporter processing and miR-9-5p production were mediated by endogenous DGCR8.

Next, we searched for human cell lines that express endogenous miR-9-5p using Daoy, U251MG, U251MG (KO), HeLa Tet-On 3G, U2OSTteOn, and HEK293TetOn3G cells. Among these, human astrocytoma U251 MG and U251 MG (KO) cells expressed high levels of miR-9-5p (Fig. 2*D*). Of importance, miR-9-5p expression levels in U251MG (KO) cells were suppressed by treatment with DGCR8 siRNAs (Fig. 2*E*), while pri-miR-9-2 was greatly increased (Fig. 2*F*). Hence, miR-9-5p production from endogenous pri-miR-9-2 occurred in a DGCR8-dependent manner. Similarly, Drosha siRNAs effectively knocked down Drosha cellular protein levels and promoted the accumulation of pri-miR-9-2 (Fig. 2, *G* and *H*). These findings suggest that the processing of both endogenous pri-miR-9-2 and pri-miR-9-2 fluorescent reporter depended on the canonical microprocessor complex.

### Pri-miR-9-2 processing is sensitive to DGCR8

**Figure 3:**
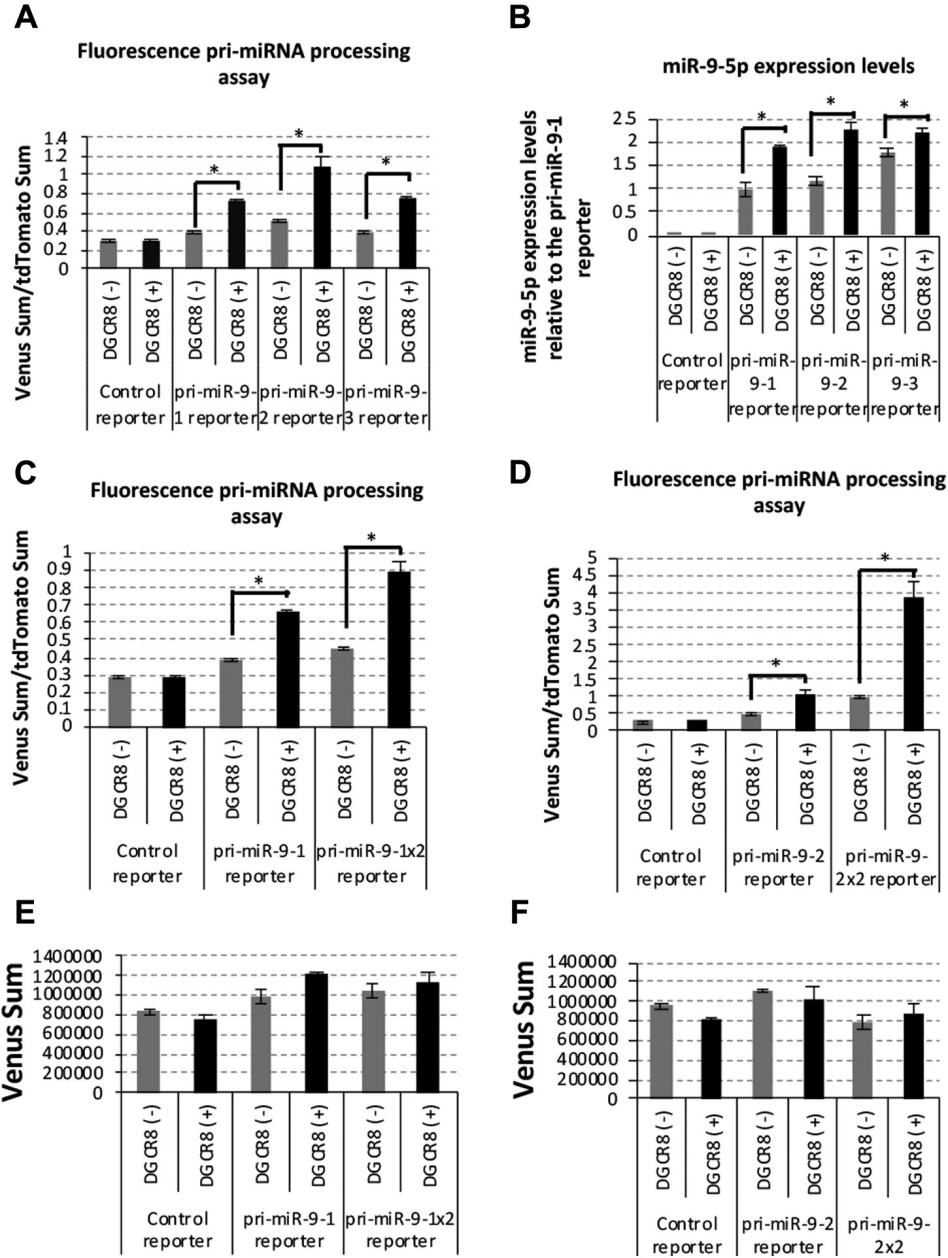
**Higher DGCR8 sensitivity of pri-miR-9-2 processing.***A*, fluorescent pri-miRNA processing reporter assay. Control, pri-miR-9-1, pri-miR-9-2, and pri-miR-9-3 were transfected with pcDNA3.1 or FLAG-DGCR8 expression vectors into HeLa Tet-On 3G cells, and fluorescent signals were monitored. The relative sum of the Venus signal intensity to the sum of the tdTomato signal intensity was calculated and shown in the graph. The error bar shows the standard deviation (n = 3). *B*, has-miR-9-5p was quantified by qRT-PCR with total RNA purified from HeLa Tet-On 3G cells transiently transfected with control, pri-miR-9-1, pri-miR-9-2, pri-miR-9-3 reporter, and FLAG-DGCR8 expression vectors. The error bar shows the standard deviation (n = 3). *C*, control, pri-miR-9-1, and pri-miR-9-1x2 (containing twice tandem repeat of pri-miR-9-1) reporter vectors were transfected with pcDNA3.1 control or DGCR8 expression vectors into HeLa Tet-On 3G cells and fluorescent signals monitored. The relative sum of the Venus signal intensity to the sum of the tdTomato signal intensity was calculated and is shown in the graph. The error bar shows the standard deviation (n = 3). *D*, control, pri-miR-9-2, and pri-miR-9-2x2 (containing twice tandem repeat of pri-miR-9-2) reporter vectors were transfected with pcDNA3.1 control or DGCR8 expression vectors into HeLa Tet-On 3G cells and fluorescent signals were monitored. The relative sum of the Venus signal intensity to the sum of the tdTomato signal intensity was calculated and is shown in the graph. The error bar shows the standard deviation (n = 3). *E* and *F*, the sum of the Venus fluorescent signal intensity in selected nuclei was calculated and is shown in the graph. The error bar shows the standard deviation (n = 3). The *asterisk* indicates significant change (*t*-test ∗*p* < 0.001).

To evaluate the efficiency of pri-miR-9-2 processing, we carried out the fluorescent miRNA processing reporter assay with pri-miR-9-1, 9-2, and 9-3 in HeLa Tet-On 3G. Among these three reporters, the pri-miR-9-2 reporter showed a relatively high efficiency of RNA processing with or without the ectopic expression of DGCR8 (Fig. 3*A*). miR-9-5p production from these reporter systems was then detected (Fig. 3*B*), and pri-miR-9-2 processing was shown to be more sensitive to DGCR8. A twice tandem repeat of the pri-miR-9-1 and pri-miR-9-2 processing reporters, pri-miR-9-1×2 and pri-miR-9-2×2, was constructed to investigate the efficiencies of pri-miRNA processing. Pri-miR-9-1×2 responded more sensitively to DGCR8 than pri-miR-9-1 without changing Venus protein levels (Fig. 3, *C* and *E*). It is surprising that pri-miR-9-2×2 reporters showed a much greater efficiency of RNA processing than pri-miR-9-1×2 and pri-miR-9-2 without changing Venus protein levels (Fig. 3, *D* and *F*). These results suggest that DGCR8-dependent pri-miRNA processing is enhanced by pri-miR-9-2.

### Identification of the DGCR8-responsive RNA element in pri-miR-9-2

**Figure 4:**
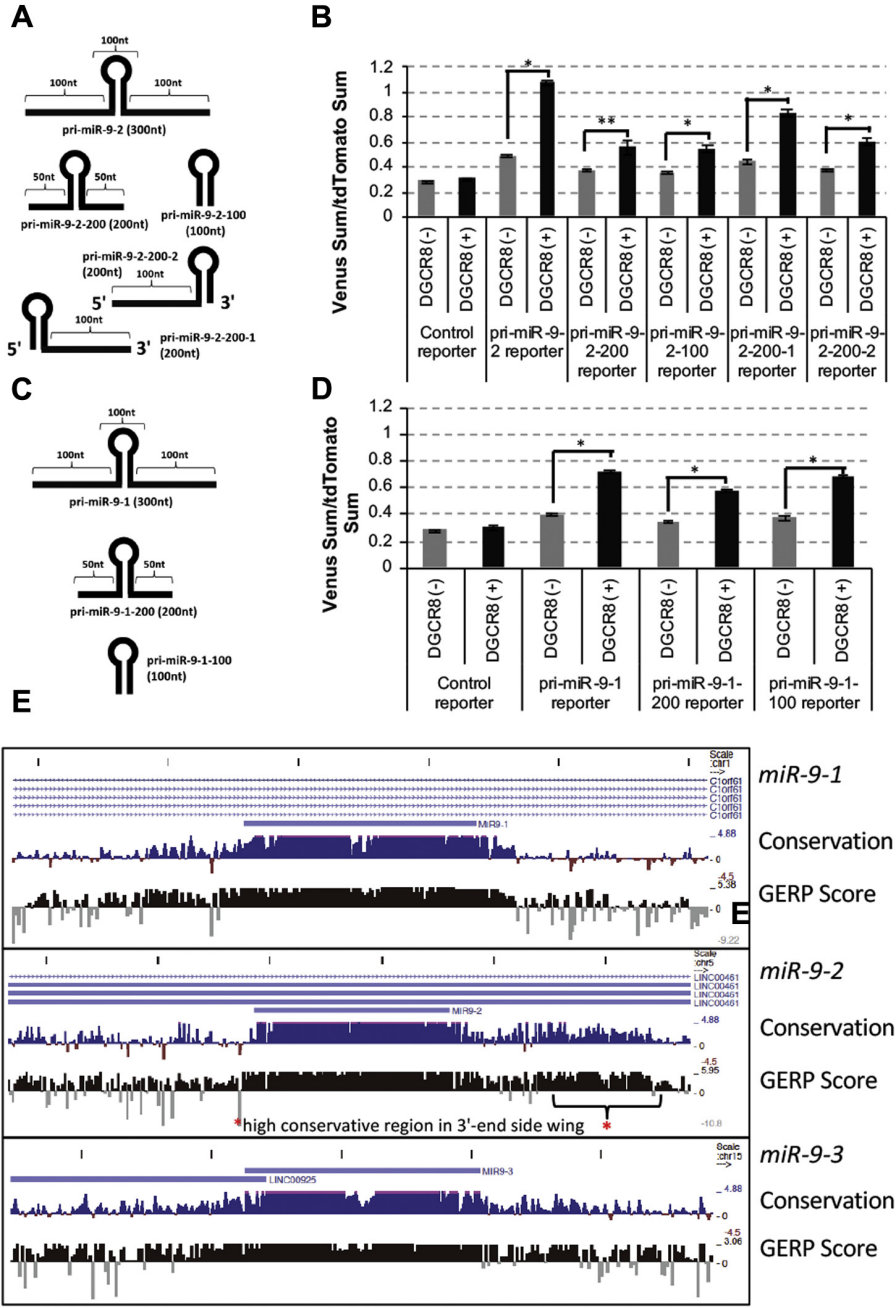
**pri-miR-9-2 has a DGCR8-responsive element in the 3’ wing region of pri-miR-9-2.***A*, schematic of deletion mutant reporters used in this study. The wing region around the stem–loop structures coding pre-miRNA was removed in the deletion mutant reporters. *B*, fluorescent pri-miRNA processing reporter assay. Control, pri-miR-9-2, pri-miR-9-2-200, pri-miR-9-2-100, pri-miR-9-2-200-1, and pri-miR-9-2-200-2 reporter vectors were transfected with pcDNA3.1 or FLAG-DGCR8 expression vectors into HeLa Tet-On 3G cells and fluorescent signals were monitored. The relative sum of the Venus signal intensity to the sum of the tdTomato signal intensity was calculated and is shown in the graph. The error bar shows the standard deviation (n = 3). *C*, schematic of deletion mutant reporters used in this study. The wing region around the stem–loop structures coding pre-miRNA was removed in the deletion mutant reporters. *D*, fluorescent pri-miRNA processing reporter assay. Control, pri-miR-9-1, pri-miR-9-1-200, and pri-miR-9-1-100 reporter vectors were transfected with pcDNA3.1 or FLAG-DGCR8 expression vectors into HeLa Tet-On 3G cells and fluorescent signals were monitored. The relative sum of the Venus signal intensity to the sum of the tdTomato signal intensity was calculated and is shown in the graph. The error bar shows the standard deviation (n = 3). *E*, alignment of human pri-miR-9-1 (300 nt) and pri-miR-9-2 (300 nt) to 35 other mammalian species by Genomic Evolutionary Rate Profiling on the UCSC Human Genome Browser. The *red asterisk* indicates the highly conserved region in the 3’-end wing of pri-miR-9-2. The *asterisk* indicates significant change (∗ *p* < 0.001 ∗∗ *p* < 0.005).

To explore key RNA elements responsible for the promotion of pri-miR-9-2 processing, we constructed various types of deletion mutant miR-9-2 (Fig. 4*A*). First, deletion mutants pri-miR-9-2-200 and pri-miR-9-2-100, which lack wing regions at both ends, showed a reduction of efficiency in HeLa Tet-On 3G, suggesting that DGCR8-responsive RNA elements are located in the wing region of pri-miR-9-2. To determine which end the elements are located in, we constructed pri-miR-9-2-200-1 and pri-miR-9-2-200-2 reporters containing 3′- and 5′-end wing sequences, respectively (Fig. 4*A*). DGCR8 sensitivity was still reduced following the transfection of pri-miR-9-2-200-2 (Fig. 4*B*), but the reduction of DGCR8 sensitivity was restored with the transfection of pri-miR-9-2-200-1 (Fig. 4*B*). This indicated that the 3′-end wing of pri-miR-9-2 contains a critical RNA element for DGCR8 sensitivity. The deletion mutant pri-miR-9-1-100, which lacks both wing regions of pri-miR-9-1, maintained its efficient pri-miRNA processing activity in a DGCR8-dependent manner, indicating the lack of any critical element in the wing region of miR-9-1 (Fig. 4, *C* and *D*).

We next performed genome alignment analysis of human pri-miR-9-2 to the sequences of 35 mammalian species from the UCSC Human Genome Browser. A highly conserved region was identified in the 3′-end wing of pri-miR-9-2, but not in the wing of pri-miR-9-1 (Fig. 4*E*). For example, a 50-nt RNA sequence in the 3′-end wing of pri-miR-9-2 was found to be 98% identical between humans and mice, compared with only 65.4% identity for a 50 nt RNA sequence in the 3′-end wing of pri-miR-9-1. This highly conserved region supports the idea that the 3′-end wing of pri-miR-9-2 contains a DRE.

**Figure 5:**
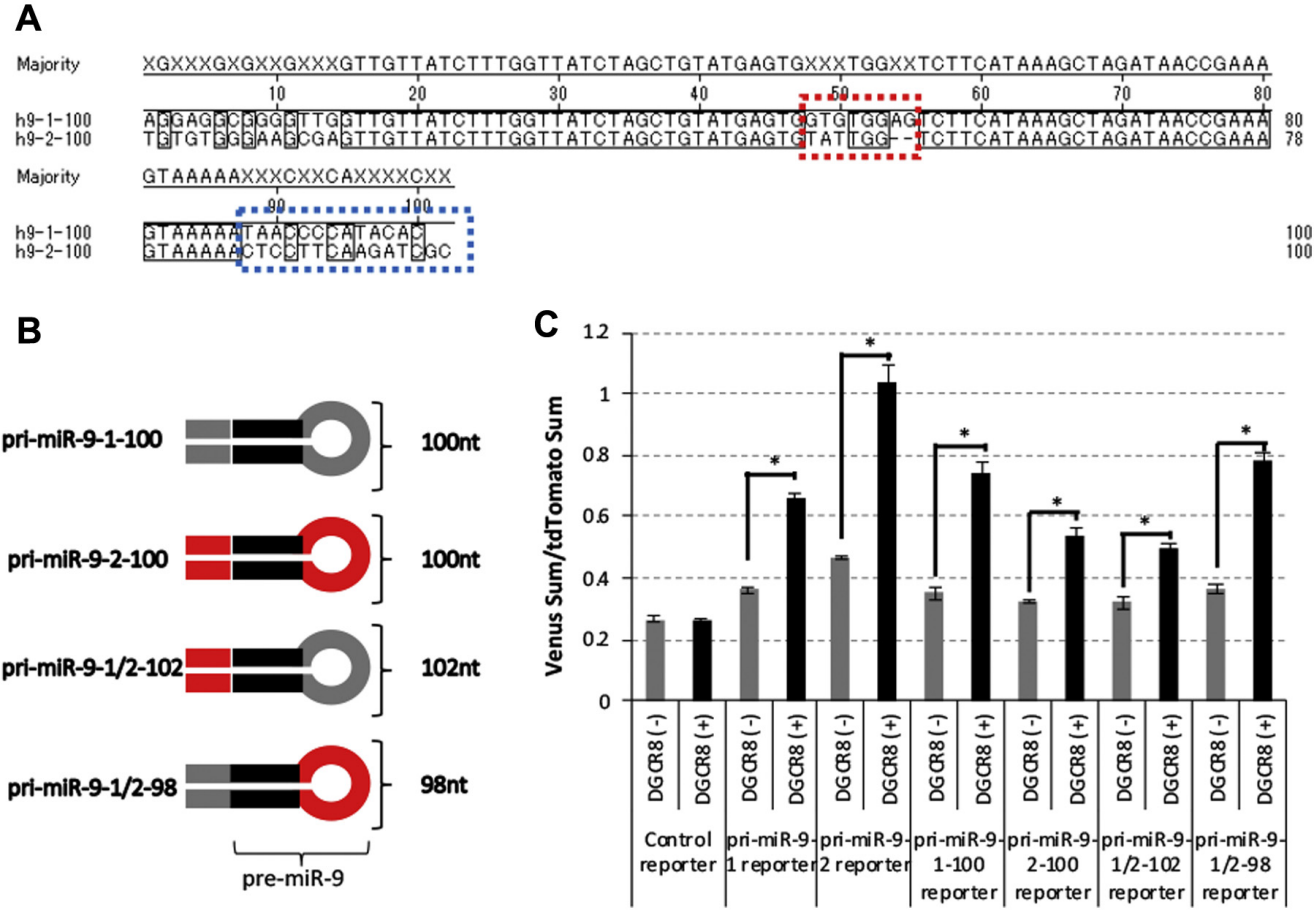
**pri-miR-9-1 also has a DGCR8-responsive element in the region near the pre-miR-9-1.***A*, alignment analysis of human pri-miR-9-1-100 and human pri-miR-9-2-100 by the Clustal W method. The 5′-end (*black dashed box*), 3′-end (*blue dashed box*), and loop between miR-9-5p and miR-9-3p (*red dashed box*) are unique. The *box* residues match the consensus/majority exactly. *B*, schematic of chimera reporters used in this study. Unique sequences of pri-miR-9-1-100 and pri-miR-9-2-100 are shown in *gray* and *red*, respectively. pri-miR-9-1/2-102 (including pre-miR-9-1, and 5′- and 3′-ends of pri-miR-9-2-100) and pri-miR-9-1/2-98 (including pre-miR-9-2, and 5′- and 3′-ends of pri-miR-9-1-100) were constructed. *C*, fluorescence pri-miRNA processing reporter assay. Control, pri-miR-9-1, pri-miR-9-2, pri-miR-9-1-100, pri-miR-9-2-100, pri-miR-9-1/2-102, and pri-miR-9-1/2-98 reporter vectors were transfected with pcDNA3.1 or FLAG-DGCR8 expression vectors and fluorescent signals were monitored. The relative sum of the Venus signal intensity to the sum of the tdTomato signal intensity was calculated and is shown in the graph. The error bar shows the standard deviation (n = 4). The *asterisk* indicates significant change (*t*-test *p* < 0.001).

**Figure 6:**
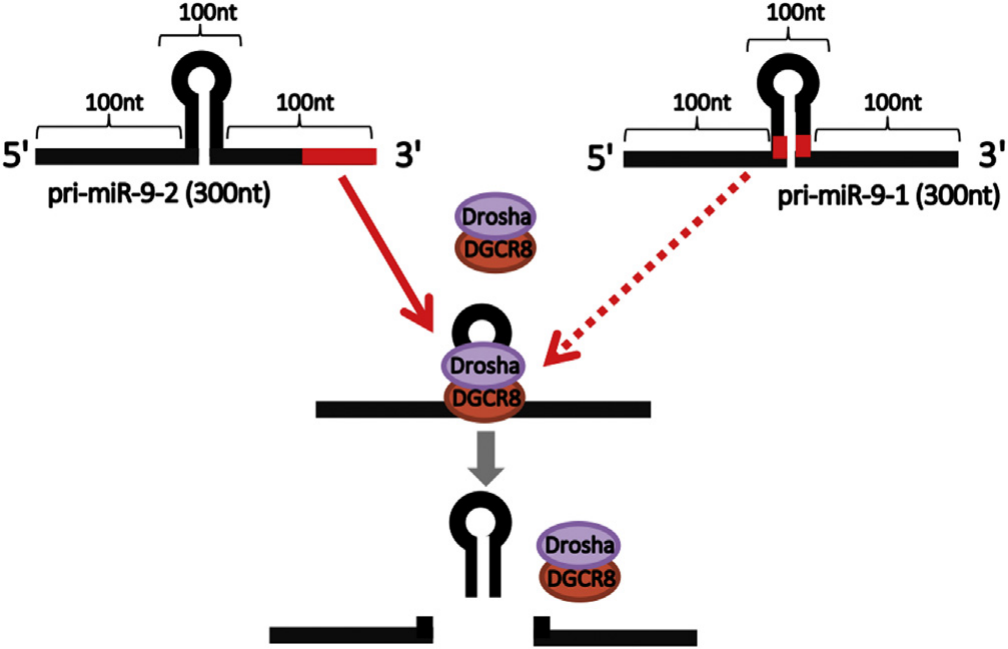
**DGCR8-responsive RNA elements in human pri-miR9-1 and pri-miR9-2.** DGCR8-responsive RNA elements (DREs) were identified in this study. The DRE of pri-miR-9-1 is in the vicinity of pre-miR-9-1, and the DRE of pri-miR-9-2 is in the 3’ wing region. DRE promotes pri-miR-9 processing activity in an ectopically expressed DGCR8-dependent manner.

We then focused on unique sequences located in the 5′-end, 3′-end, and loop region between miR-9-5p and miR-9-3p in pri-miR-9-1-100 (Fig. 5*A*). To explore pri-miR-9-1-100 sequences responsible for potentiating DGCR8-dependent processing, we constructed fluorescence reporter vectors including pri-miR-9-1-100 and pri-miR-9-2-100 chimeras, pri-miR-9-1/2-102 (including pre-miR-9-1, and 5′- and 3′-ends of pri-miR-9-2-100), and pri-miR-9-1/2-98 (including pre-miR-9-2, and 5′- and 3′-ends of pri-miR-9-1-100) (Fig. 5*B*). Reporter analysis revealed that an RNA element containing 5′- and 3′-end sequences of pri-miR-9-1-100 showed higher DGCR8 sensitivity than pri-miR-9-2-100 (Fig. 5*C*). This suggested that 5′- and 3′-end sequences of pri-miR-9-1-100, but not the unique loop sequence of pre-miR-9-1, contain important elements for high sensitivity to DGCR8. Collectively, we identified novel DREs in pri-miR-9-1 and pri-miR-9-2. In pri-miR-9-1, RNA sequences close to the 5′- and 3′-ends of pre-miR-9-1 appeared to be important, whereas the 3′-end wing was important in pri-miR-9-2 (Fig. 6).

### Exploration of pri-miRNA candidates possessing DRE

**Figure 7:**
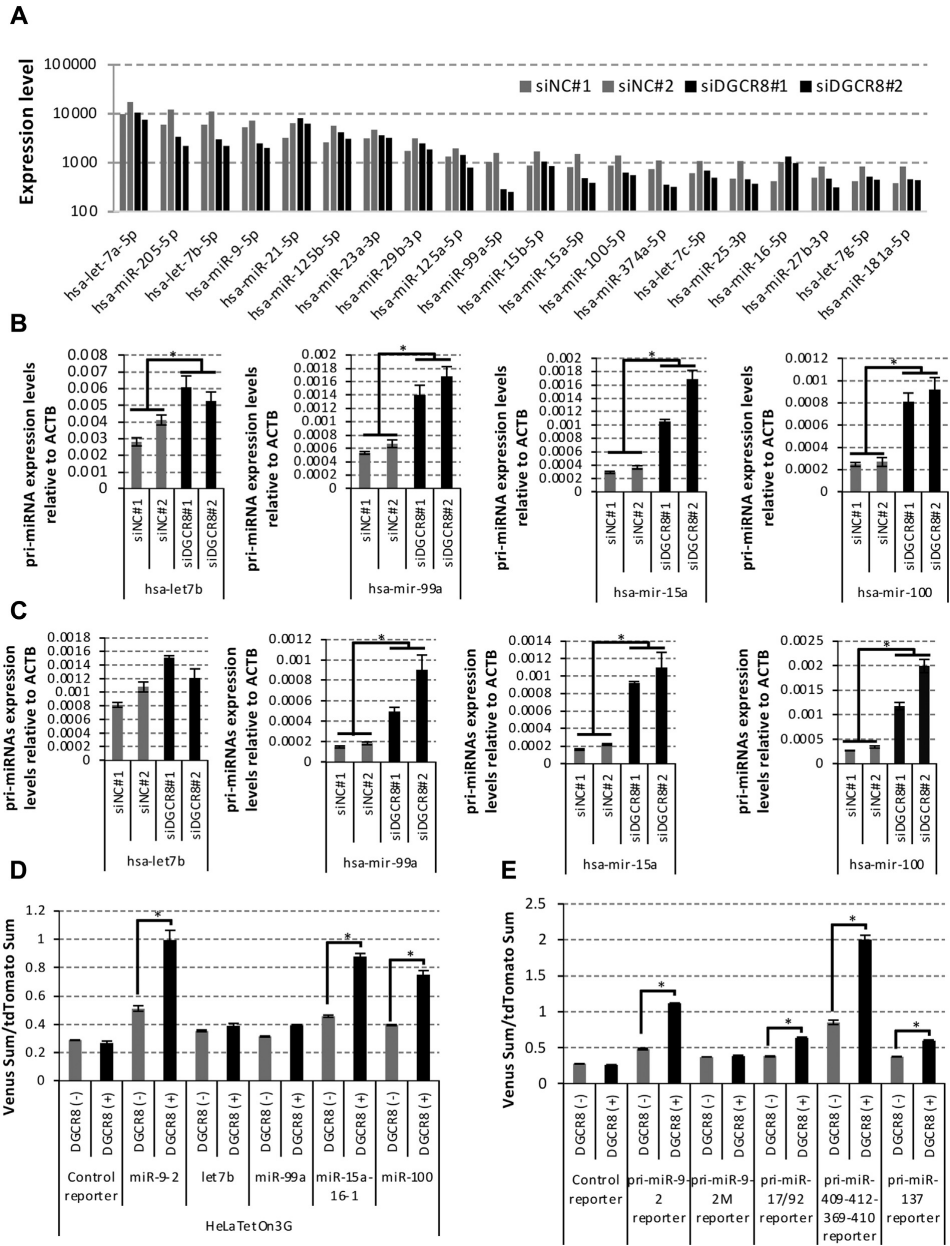
**Exploration of pri-miRNA candidates possessing DRE.***A*, miRNA profiling of U251 MG (KO) cells treated with siNC#1, siNC#2 siDGCR8#1, and siDGCR8#2 was performed with the nCounter miRNA analysis system. Expression levels of top20 ranked miRNAs are shown in the graph. The bar graph indicates the average in each two technical replicates (n = 1). *B*, pri-miRNA expression levels of hsa-let-7b-5p, hsa-miR-99a-5p, hsa-miR-15a-5p, and hsa-miR-100-5p in U251 MG (KO) cells treated with siNC#1, siNC#2 siDGCR8#1, and siDGCR8#2 were quantified by qRT-PCR. The error bar represents SD using four biological replicates. *C*, pri-miRNA expression levels of hsa-let-7b-5p, hsa-miR-99a-5p, hsa-miR-15a-5p, and hsa-miR-100-5p in HeLa Tet-On 3G cells treated with siNC#1, siNC#2 siDGCR8#1, and siDGCR8#2 were quantified by qRT-PCR. The error bar represents SD using four biological replicates. *D*, pri-miRNA processing reporters containing pri-miR-9-2 (300 nt), pri-let-7b (300 nt), pri-miR-99a (300 nt), pri-miR-15a-16-1 (400 nt), and pri-miR-100 (300 nt) were constructed and the processing assay was performed with HeLa Tet-On 3G cells. The error bar represents SD using three biological replicates. *E*, pri-miRNA processing reporters containing pri-miR-9-2 (300 nt), pri-miR-9-2M (300 nt), pri-miR-17/92 (887 nt), pri-miR-409-412-369-410 (800 nt), and pri-miR-137 (500 nt) were constructed, and the processing assay was performed with HeLa Tet-On 3G cells. The error bar represents SD using three biological replicates. *Asterisk*s indicate significant change (∗*p* < 0.01).

To explore pri-miRNA candidates possessing DRE, we conducted a BLAST search using the highly conserved 50-nt RNA sequence in the 3′-end wing of pri-miR-9-2 but found no matching transcript in the human transcriptome. We performed miRNA profiling to explore DGCR8-sensitive miRNA candidates in U251 MG (KO) cells treated with DGCR8 siRNA using the nCounter Analysis system (Fig. 7*A*). Of importance, qRT-PCR assay confirmed accumulation of several pri-miRNAs, including pri-miR-99a, pri-miR-15a, and pri-miR-100, in both U251 MG (KO) and HeLa Tet On 3G cells treated with DGCR8 siRNAs (Fig. 7, *B* and *C*). Our reporter system also revealed that pri-miR-15a-16-1 and pri-miR-100 displayed efficient processing in an ectopically expressing DGCR8-dependent manner but that this was less efficient than the pri-miR-9-2 reporter (Fig. 7*D*).

Schizophrenia and 22q11.2 deletion syndrome–related miRNAs, miR-17-92 cluster pri-miRNA, miR-409-412-369-410 cluster pri-miRNA, and miR-137 were also investigated (34, 35, 36, 37). miR-17-92 cluster pri-miRNA, which contains six different pre-miRNAs, and pri-miR-137 reporters showed less efficient processing than the pri-miR-9-2 reporter (Fig. 7*E*). The miR-409-412-369-410 cluster pri-miRNA reporter showed less efficient processing than the pri-miR-9-2×2 reporter, even though it contained four different pre-miRNAs (Fig. 7*E*).

### Role of DRE in DSCR8-dependent microprocessor activity

**Figure 8:**
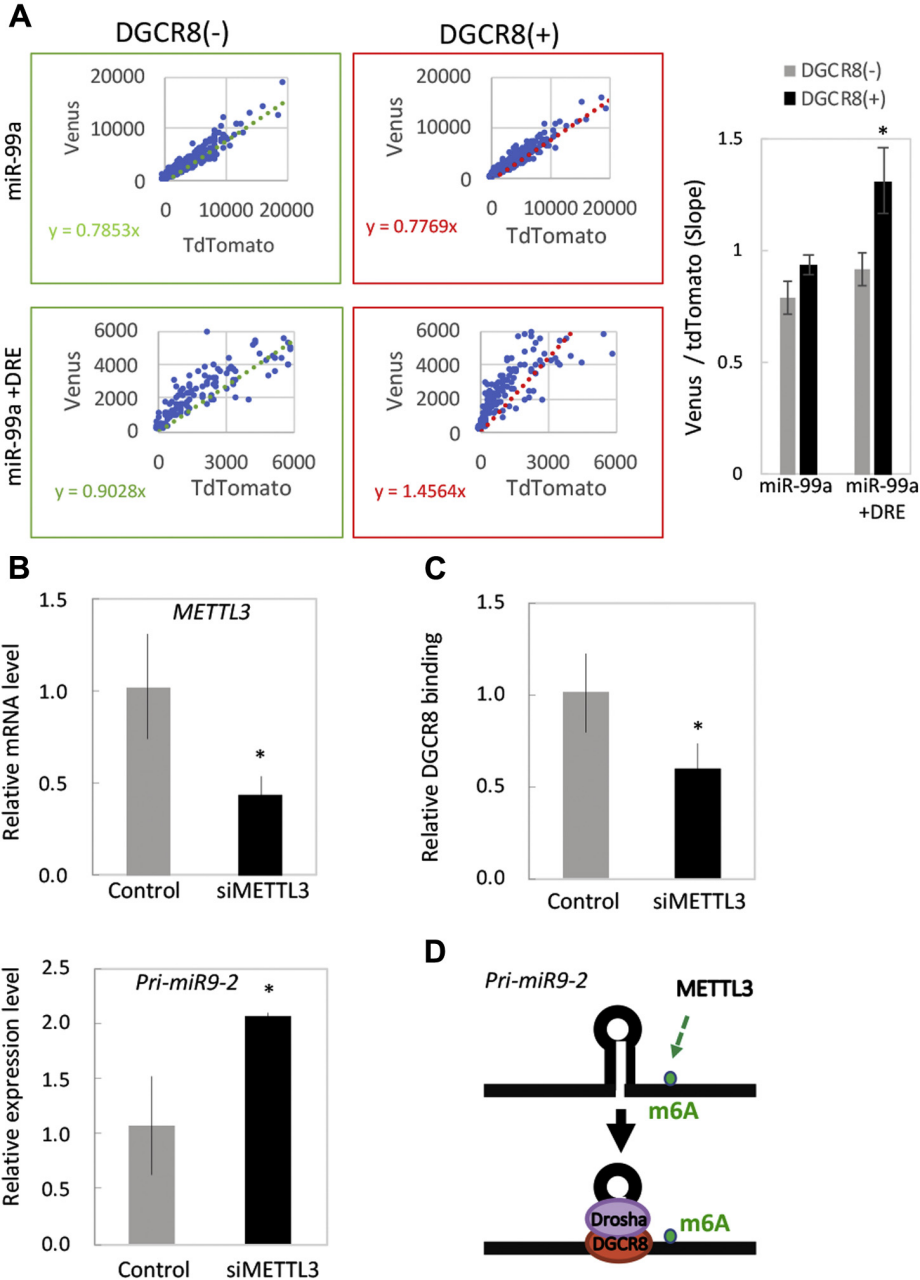
**Role of DRE in DGCR8-dependent microprocessor activity.***A*, pri-miRNA processing reporters containing pri-miR-99a (300 nt) and pri-miR-99a+DRE (300 nt) were constructed, and the processing assay was performed with HeLa Tet-On 3G cells. The graph indicates plots for each cell obtained from Venus and tdTomato fluorescence signals. Slopes from linear regression are shown in each graph. The bar graph indicates slopes with or without DGCR8. The *asterisk* indicates significant change (*t*-test *p* = 0.026). *B*, RNA expression levels of METTL3 and unprocessed pri-miR-9-2 in U251 MG (KO) cells treated with siNC#1, siNC#2 siMETTL3#1, and siMETTL3#2 were quantified by qRT-PCR. The *asterisk* indicates significant change (*t*-test *p* < 0.01) *C*, Quantification of DGCR8-bound pri-miRNA was analyzed by CLIP-qRT-PCR assay. The *asterisk* indicates significant change (*t*-test *p* < 0.05). *D*, Model of DRE in DGCR8-dependent microprocessor activity. DRE, DGCR8-responsive RNA element; CLIP, UV cross-linking and immunoprecipitatio.

Among the DGCR8-sensitive miRNAs we found, the miR-99a reporter does not respond to DGCR8 expression (Fig. 7*D*), whereas DGCR8 knockdown upregulates pri-miR-99a and downregulates mature miR-99a (Fig. 7, *A*–*C*). Therefore, we tried to add a chimeric reporter containing the 3’ wing DRE of miR-9-2 in miR-99a reporter to test whether DGCR8 sensitivity depends on the DRE sequence. Of interest, a simple conversion of the 3’ wing region in miR-99a reporter to a DRE sequence increased DGCR8 response (Fig. 8*A*). On the other hand, control and miR-99a reporter did not show DGCR8 responsiveness (Figs. 8*A* and S2). These results strongly suggest the functional significance of DGCR8 sensitivity in the DRE sequence. Since the DRE sequence itself did not show a double-stranded structure, RNA duplex among typical pri-miRNAs (Fig. S3), we considered the involvement of RNA modification and DGCR8 sensitivity. Given that an RNA modification with N6-methyladenosine is efficiently recognized by a microprocessor complex (38), we performed a knockdown experiment of METTL3, an epi-transcriptome m6A writer enzyme in U251MG(KO) cells. Similar to previous study, we also confirmed upregulation of pri-miR-9-2 expression in METTL3 KD cells (38) (Fig. 8*B*). By UV cross-linking and immunoprecipitation (CLIP)-qRT-PCR assay, we observed that the efficiency of interaction between DGCR8 and pri-miR-9-2 was decreased in METTL3 KD cells (38) (Fig. 8*C*). These results suggest that the DGCR8 sensitivity by adding the DRE sequence depends on RNA modification and DGCR8 accessibility (Fig. 8*D*).

Taken together, our findings show that the pri-miR-9-2 reporter had the most efficient processing of all pri-miRNA reporters investigated in this study and that the DRE identified in pri-miR-9-2 might be a unique RNA element for potentiating DGCR8-dependent processing.

## Discussion

In the present study, we found that pri-miR-9 is the transcript most efficiently processed out of other miRNA species by the canonical microprocessor complex. We also identified novel sensitive RNA elements using a modified version of the previously described *in vivo* pri-miRNA processing fluorescence reporter system (31). This reporter system enabled the investigation of DGCR8- and Drosha-dependent microprocessor activity and efficiency by observation of a ratiometric fluorescence color reporter, which mimicked the cleavage process from pri-miRNA to pre-miRNA and mature miRNA.

Previous studies reported that human pri-miR-9-2 is efficiently processed in a DGCR8-dependent manner in 22q11.2 deletion syndrome model mice and Dgcr8-deficient mice (29, 34). These mice also displayed a psychiatric phenotype and showed an accrual of pri-miR-9-2, which was one of the most accumulated pri-miRNAs in the prefrontal cortex and hippocampus. Therefore, miR-9 is thought to be a strong candidate to understand the molecular etiology of schizophrenia and other neurological diseases. Indeed, miR-9 regulates neurogenesis in the mouse telencephalon by targeting downstream mRNAs, as evidenced by a study of miR-9 knockout mice (39). miR-9/9∗ are also able to directly convert adult human fibroblasts to neurons through the control of chromatin accessibility by inhibiting neuron-restricted silencer factor (40). Given the two-hit hypothesis for schizophrenia, including a combination of genetic and environmental factors (41) and quality control of neurogenic factors, miR-9 must be critically involved at multiple RNA steps.

In this study, we provide evidence that pri-miR-9-2 is processed by the canonical microprocessor complex including DGCR8 and Drosha, that the pri-miR-9-2 processing efficiency is relatively high, and that a mediating DRE sequence is present in the 3′-end wing of pri-miR-9-2. Of interest, this DRE is highly conserved among mammals, supporting the idea that it is a critical quality control element. We also found other DRE sequences near both ends of pre-miR-9-1 that are important for DGCR8 sensitivity. Of importance, conservation of these RNA sequences between humans and mice is 100% identical, while the comparable region in pri-miR-9-2-100 is not (Fig. 6). Recently, an RNA modification with N6-methyladenosine functions was reported as a marker that is efficiently recognized by a microprocessor complex (38, 42). It is possible that the DREs in the present study have the potential to be similarly methylated. In fact, a recent m6A HIT-CLIP result derived from mouse brain shows two independent m6A methylation sites in the 3’ wing region on pri-miR-9-2 (43) (Fig. S4). Given the high interspecies conservation of this 3’ wing region, this DRE might be deeply involved with DGCR8 sensitivity.

Among the pri-miRNA candidates we explored for possessing DRE to potentiate pri-miRNA processing, the pri-miR-9-2 reporter showed the most efficient processing. This indicated that the DRE RNA element of pri-miR-9-2 may be unique for potentiating DGCR8-dependent processing and that it might be required for pursuing the link to schizophrenia in the future.

## Experimental procedures

### Vector construction and plasmid preparation

Vector construction was performed by GENEWIZ. Synthesized FLAG-human-DGCR8 and FLAG-tagged DGCR8 mutant with the C-terminal tail (701–773 aa) deleted were subcloned into the *Hind*III/*Not*I site of pcDNA3.1(+) vectors (GenBank:CAK54796.1). pri-miRNA processing reporters were engineered based on the bidirectional tetracycline-inducible vector, pTRE-Tight-BI. Synthesized Venus cDNA with an N-terminal nuclear localization signal and C-terminal PEST cDNA were subcloned into the *Eco*R I/*Xba* I site in MSC-II of the pTRE-Tight-BI vector, and tdTomato cDNA with an N-terminal nuclear localization signal, C-terminal PEST, and pri-miRNA cDNA were subcloned into the *Xma* I/*Cla* I site in MCS I of the pTRE-Tight-BI vector. The detailed sequences are shown in Table S1.

### Cell culture and transfection

HeLa Tet-On 3G, U2OS Tet-On, and HEK293 Tet-On 3G cell lines were purchased from Takara-Clontech. Daoy, U251 MG, and U251 MG (KO) cell lines were purchased from JCRB Cell Bank. HeLa Tet-On 3G cells were cultured in FluoroBrite Dulbecco's modified Eagle's medium (Life Technologies) with 10% fetal bovine serum (Life Technologies) in 5% CO_2_ at 37 °C for maintenance. For the fluorescence reporter assay, HeLa Tet-On 3G cells were cultured with a 10% Tet-system–approved fetal bovine serum (Clontech) in 5% CO_2_ at 37 °C. Cells were transfected with Lipofectamine3000 (Life Technologies) and Opti-MEMI (ThermoFisher Scientific) following the manufacturer’s instructions. For transfection of the reporter and/or expression vectors, 1× 10^4^ cells were seeded in CellCarrier-96 plates (PerkinElmer) or 1.6 × 10^5^ cells were seeded in 6-well plates (Corning). Three hours after transfection, the culture medium containing transfection reagents was changed to fresh medium containing 10 ng/ml doxycycline. Nonconfocal images of the cells were obtained 18 to 24 h later by the Opera Phenix high-content screening system with a 10× Air, NA0.3 objective lens (PerkinElmer).

### Measurement of live-cell pri-miRNA processing activity

Venus and tdTomato signal intensities were obtained for 20 and 5 ms, respectively. Images were obtained from eight fields of view for each well, and nuclei with Venus signals were selected by the NEW Opera Phenix HCS System (PerkinElmer). The sum of the Venus and tdTomato signals in each well was calculated from the Venus and tdTomato signal intensities of each cell in the well. The relative sum of the Venus signal intensity to the sum of the tdTomato signal intensity in each well was calculated as the pri-miRNA processing activity and relative values are shown in the graph. Alternatively, the cell images were obtained by KEYENCE BZ-X810. Quantification of Venus and tdTomato fluorescence signal from single cells were automatically quantified using the Hybrid Cell Count Module BZ-H4C (KEYENCE). The slope of the Venus and tdTomato signals as the pri-miRNA processing activity is shown in the graph.

### siRNA transfection

For siRNA transfection, HeLa Tet-On 3G cells (Takara Bio) or U251MGKO cells (JCRB Cell Bank) were seeded in CellCarrier-96 plates (PerkinElmer) or 6-well plates (Corning). siRNAs were transfected with Lipofectamine RNAiMAX Transfection Reagent (ThermoFisher Scientific) in Opti-MEMI following the manufacturer’s instructions. siRNAs purchased from Invitrogen in this study are listed below.

siNC#1 as a negative control siRNA in human, mouse, and rat cells;

siNC#2 as a negative control siRNA in human, mouse, and rat cells;

siDGCR8#1, a human DGCR8 siRNA

sense sequence (5′–3′), CCCUGUCUAUAAUUUCUUUtt

antisense sequence (5′–3′), AAAGAAAUUAUAGACAGGGcg;

siDGCR8#2, a human DGCR8 siRNA

sense sequence (5′–3′), GGAUCAUGACAUUCCAUAAtt

antisense sequence (5′–3′), UUAUGGAAUGUCAUGAUCCac;

siDGCR8#3, a human DGCR8 siRNA

sense sequence (5′–3′), GGUUCACGGCUAAAGCAAUtt

antisense sequence (5′–3′), AUUGCUUUAGCCGUGAACCcg;

siDrosha#1, a human Drosha/RNASEN siRNA

sense sequence (5′–3′), GCUCUGUCCGUAUCGAUCAtt

antisense sequence (5′–3′),

UGAUCGAUACGGACAGAGCtt;

siDrosha#2, a human Drosha/RNASEN siRNA

sense sequence (5′–3′), GACCAGACUUUGUACCCUUtt

antisense sequence (5′–3′), AAGGGUACAAAGUCUGGUCgt;

siDrosha#3, a human Drosha/RNASEN siRNA

sense sequence (5′–3′), CACUUAACUUUGUUGCGAAtt

antisense sequence (5′–3′), UUCGCAACAAAGUUAAGUGtc

siMettl3#1, a human Drosha/RNASEN siRNA

sense sequence (5′–3′), GAUCCUGAGUUAGAGAAGAtt

antisense sequence (5′–3′),

UCUUCUCUAACUCAGGAUtg;

siMettl3#2, a human Drosha/RNASEN siRNA

sense sequence (5′–3′),

GCAGUUCCUGAAUUAGCUAtt

antisense sequence (5′–3′),

UAGCUAAUUCAGGAACUGCtg;

### Quantification of miRNA, pri-miRNA, and mRNA by quantitative RT-PCR

Total RNAs were extracted using the miRNeasy Mini kit (Qiagen) and quantified by Qubit with the Qubit RNA HS Assay Kit (Life Technologies). Quantification of hsa-miR-9-5p and hsa-miR-9-3p were performed with a TaqMan microRNA RT Kit (Life Technologies, TM/RT000583 and TM/RM002231, respectively), TaqMan microRNA Assays (Life Technologies), and TaqMan Universal PCR Master Mix II with UNG (Life Technologies) following the manufacturer’s instructions. Five nanograms of total RNA was used in each reverse transcription reaction. To quantify human *DGCR8* mRNA, human *Drosha* mRNA, and human β-actin mRNA expression levels, real-time PCR was performed using the TaqMan Gene Expression assay (Life Technologies, *DGCR8*; Hs00256062_m1 and Hs00987085_m1, *Drosha*; Hs00203008_m1, β-actin; Hs01060665_g1, pri-let-7b; Hs03302548-pri, pri-miR-100; Hs03302731-pri, pri-miR-15a; Hs03302582-pri, pri-miR-9-1; Hs03303201_pri, pri-miR-9-2; Hs03303202_pri, pri-miR-9-3; Hs03293595_pri, pri-miR-99a; Hs03302729-pri), TaqMan Gene Expression Master Mix (Life Technologies), and ViiA7 Real Time PCR or StepOnePlus system (Life Technologies) following the manufacturer’s instructions. Quantitative RT-PCR analysis was used with at least three biological replicates.

### Western blotting with the Wes system

Samples prepared for western blotting underwent simple western analysis with the Wes system and 12–230 kDa Wes Separation Module (Protein Simple) using rabbit polyclonal anti-DGCR8 (PGI Proteintech Group), rabbit polyclonal anti-Drosha (Bethyl Laboratories), and anti-β-actin (Novus Biologicals) antibodies. Data analysis was performed with Compass software (Protein Simple).

### miRNA profiling with an nCounter miRNA analysis system

miRNA profiling analysis of total RNA was performed with an nCounter Analysis System and a Human miRNA Assay Kit Version 3.0 (NanoString Technologies) using the FOV max mode. Data analyses were performed with nSolver analysis software version 2.0 equipped with the nCounter Analysis System. Normalization of miRNA profiling data was performed with housekeeping gene expression levels. This experiment was carried out in two technical replicates from one each sample.

### Genomic Evolutionary Rate Profiling

The Genomic Evolutionary Rate Profiling score defined the reduction in the number of substitutions among the multispecies sequence alignment, using 35 other mammalian species to human genome (44,45). All these data analyses were done using the publicly available tool, the UCSC Genome Browser (https://genome.ucsc.edu).

### CLIP-RT-qPCR assay

The CLIP-RT-qPCR assay was performed as described (46).U251 cells were UV cross-linked at 254 nm (UV-B) with 200 mJ/cm^2^ three times. Lysates were subjected to immunoprecipitation and qRT-PCR (47). CLIP-qRT-PCR enrichments were normalized by quantifying relative levels of immunoprecipitated pri-miRNA to input from lysates. The detailed primer sequences are shown in Table S2.

### Statistical analyses

All experiments were carried out using at least three biological replicates. Statistically significant differences were calculated by two-tailed Student’s *t*-test and presented as the mean and standard deviation.

## Data availability

All data are contained within this article and supplemental information.

## Supporting information

This article contains supporting information.

## Conflict of interest

H. O. is a paid member of the Scientific Advisory Board of San Bio Co, Ltd and K Pharma, Inc M. Y. is a scientific advisor of K Pharma, Inc.

## References

[1] Ambros V. (2011). MicroRNAs and developmental timing. Curr. Opin. Genet. Dev..

[2] Guo H., Ingolia N.T., Weissman J.S., Bartel D.P. (2010). Mammalian microRNAs predominantly act to decrease target mRNA levels. Nature.

[3] Fabian M.R., Sonenberg N., Filipowicz W. (2010). Regulation of mRNA translation and stability by microRNAs. Annu. Rev. Biochem..

[4] Kim V.N., Han J., Siomi M.C. (2009). Biogenesis of small RNAs in animals. Nat. Rev. Mol. Cell Biol..

[5] Lee Y., Jeon K., Lee J.T., Kim S., Kim V.N. (2002). MicroRNA maturation: Stepwise processing and subcellular localization. EMBO J..

[6] Lee Y., Ahn C., Han J., Choi H., Kim J., Yim J., Lee J., Provost P., Rådmark O., Kim S., Kim V.N. (2003). The nuclear RNase III Drosha initiates microRNA processing. Nature.

[7] Denli A.M., Tops B.B., Plasterk R.H., Ketting R.F., Hannon G.J. (2004). Processing of primary microRNAs by the Microprocessor complex. Nature.

[8] Gregory R.I., Yan K.P., Amuthan G., Chendrimada T., Doratotaj B., Cooch N., Shiekhattar R. (2004). The Microprocessor complex mediates the genesis of microRNAs. Nature.

[9] Han J., Lee Y., Yeom K.H., Kim Y.K., Jin H., Kim V.N. (2004). The Drosha-DGCR8 complex in primary microRNA processing. Genes Dev..

[10] Landthaler M., Yalcin A., Tuschl T. (2004). The human DiGeorge syndrome critical region gene 8 and its D. melanogaster homolog are required for miRNA biogenesis. Curr. Biol..

[11] Hutvágner G., McLachlan J., Pasquinelli A.E., Bálint E., Tuschl T., Zamore P.D. (2001). A cellular function for the RNA-interference enzyme Dicer in the maturation of the let-7 small temporal RNA. Science.

[12] Bernstein E., Caudy A.A., Hammond S.M., Hannon G.J. (2001). Role for a bidentate ribonuclease in the initiation step of RNA interference. Nature.

[13] Blahna M.T., Hata A. (2013). Regulation of miRNA biogenesis as an integrated component of growth factor signaling. Curr. Opin. Cell Biol..

[14] Croce C.M. (2009). Causes and consequences of microRNA dysregulation in cancer. Nat. Rev. Genet..

[15] Cao X., Yeo G., Muotri A.R., Kuwabara T., Gage F.H. (2006). Noncoding RNAs in the mammalian central nervous system. Annu. Rev. Neurosci..

[16] Lagos-Quintana M., Rauhut R., Yalcin A., Meyer J., Lendeckel W., Tuschl T. (2002). Identification of tissue-specific microRNAs from mouse. Curr. Biol..

[17] Krichevsky A.M., King K.S., Donahue C.P., Khrapko K., Kosik K.S. (2003). A microRNA array reveals extensive regulation of microRNAs during brain development. RNA.

[18] Deo M., Yu J.Y., Chung K.H., Tippens M., Turner D.L. (2006). Detection of mammalian microRNA expression by *in situ* hybridization with RNA oligonucleotides. Dev. Dyn..

[19] Kapsimali M., Kloosterman W.P., de Bruijn E., Rosa F., Plasterk R.H., Wilson S.W. (2007). MicroRNAs show a wide diversity of expression profiles in the developing and mature central nervous system. Genome Biol..

[20] Yuva-Aydemir Y., Simkin A., Gascon E., Gao F.B. (2011). MicroRNA-9: Functional evolution of a conserved small regulatory RNA. RNA Biol..

[21] Davila J.L., Goff L.A., Ricupero C.L., Camarillo C., Oni E.N., Swerdel M.R., Toro-Ramos A.J., Li J., Hart R.P. (2014). A positive feedback mechanism that regulates expression of miR-9 during neurogenesis. PLoS One.

[22] Delaloy C., Liu L., Lee J.A., Su H., Shen F., Yang G.Y., Young W.L., Ivey K.N., Gao F.B. (2010). MicroRNA-9 coordinates proliferation and migration of human embryonic stem cell-derived neural progenitors. Cell Stem Cell.

[23] Miller J.A., Ding S.L., Sunkin S.M., Smith K.A., Ng L., Szafer A., Ebbert A., Riley Z.L., Royall J.J., Aiona K., Arnold J.M., Bennet C., Bertagnolli D., Brouner K., Butler S. (2014). Transcriptional landscape of the prenatal human brain. Nature.

[24] Hauberg M.E., Roussos P., Grove J., Børglum A.D., Mattheisen M., Schizophrenia, W. G. O. T. P. G. C. (2016). Analyzing the role of MicroRNAs in schizophrenia in the Context of Common genetic risk variants. JAMA Psychiatry.

[25] Shiohama A., Sasaki T., Noda S., Minoshima S., Shimizu N. (2003). Molecular cloning and expression analysis of a novel gene DGCR8 located in the DiGeorge syndrome chromosomal region. Biochem. Biophys. Res. Commun..

[26] Bassett A.S., McDonald-McGinn D.M., Devriendt K., Digilio M.C., Goldenberg P., Habel A., Marino B., Oskarsdottir S., Philip N., Sullivan K., Swillen A., Vorstman J., International D.S.C. (2011). Practical guidelines for managing patients with 22q11.2 deletion syndrome. J. Pediatr..

[27] Pulver A.E., Nestadt G., Goldberg R., Shprintzen R.J., Lamacz M., Wolyniec P.S., Morrow B., Karayiorgou M., Antonarakis S.E., Housman D. (1994). Psychotic illness in patients diagnosed with velo-cardio-facial syndrome and their relatives. J. Nerv Ment. Dis..

[28] Murphy K.C., Jones L.A., Owen M.J. (1999). High rates of schizophrenia in adults with velo-cardio-facial syndrome. Arch. Gen. Psychiatry.

[29] Stark K.L., Xu B., Bagchi A., Lai W.S., Liu H., Hsu R., Wan X., Pavlidis P., Mills A.A., Karayiorgou M., Gogos J.A. (2008). Altered brain microRNA biogenesis contributes to phenotypic deficits in a 22q11-deletion mouse model. Nat. Genet..

[30] Ouchi Y., Banno Y., Shimizu Y., Ando S., Hasegawa H., Adachi K., Iwamoto T. (2013). Reduced adult hippocampal neurogenesis and working memory deficits in the Dgcr8-deficient mouse model of 22q11.2 deletion-associated schizophrenia can be rescued by IGF2. J. Neurosci..

[31] Weitz S.H., Gong M., Barr I., Weiss S., Guo F. (2014). Processing of microRNA primary transcripts requires heme in mammalian cells. Proc. Natl. Acad. Sci. U. S. A..

[32] Faller M., Toso D., Matsunaga M., Atanasov I., Senturia R., Chen Y., Zhou Z.H., Guo F. (2010). DGCR8 recognizes primary transcripts of microRNAs through highly cooperative binding and formation of higher-order structures. RNA.

[33] Yeom K.H., Lee Y., Han J., Suh M.R., Kim V.N. (2006). Characterization of DGCR8/Pasha, the essential cofactor for Drosha in primary miRNA processing. Nucleic Acids Res..

[34] Fénelon K., Xu B., Lai C.S., Mukai J., Markx S., Stark K.L., Hsu P.K., Gan W.B., Fischbach G.D., MacDermott A.B., Karayiorgou M., Gogos J.A. (2013). The pattern of cortical dysfunction in a mouse model of a schizophrenia-related microdeletion. J. Neurosci..

[35] Toyoshima M., Akamatsu W., Okada Y., Ohnishi T., Balan S., Hisano Y., Iwayama Y., Toyota T., Matsumoto T., Itasaka N., Sugiyama S., Tanaka M., Yano M., Dean B., Okano H. (2016). Analysis of induced pluripotent stem cells carrying 22q11.2 deletion. Transl. Psychiatry.

[36] Sakamoto K., Crowley J.J. (2018). A comprehensive review of the genetic and biological evidence supports a role for MicroRNA-137 in the etiology of schizophrenia. Am. J. Med. Genet. B Neuropsychiatr. Genet..

[37] Cheng Y., Wang Z.M., Tan W., Wang X., Li Y., Bai B., Li Y., Zhang S.F., Yan H.L., Chen Z.L., Liu C.M., Mi T.W., Xia S., Zhou Z., Liu A. (2018). Partial loss of psychiatric risk gene Mir137 in mice causes repetitive behavior and impairs sociability and learning via increased Pde10a. Nat. Neurosci..

[38] Alarcón C.R., Lee H., Goodarzi H., Halberg N., Tavazoie S.F. (2015). N6-methyladenosine marks primary microRNAs for processing. Nature.

[39] Shibata M., Nakao H., Kiyonari H., Abe T., Aizawa S. (2011). MicroRNA-9 regulates neurogenesis in mouse telencephalon by targeting multiple transcription factors. J. Neurosci..

[40] Abernathy D.G., Kim W.K., McCoy M.J., Lake A.M., Ouwenga R., Lee S.W., Xing X., Li D., Lee H.J., Heuckeroth R.O., Dougherty J.D., Wang T., Yoo A.S. (2017). MicroRNAs Induce a Permissive chromatin environment that Enables neuronal Subtype-specific Reprogramming of adult human fibroblasts. Cell Stem Cell.

[41] Caspi A., Moffitt T.E. (2006). Gene-environment interactions in psychiatry: Joining forces with neuroscience. Nat. Rev. Neurosci..

[42] Alarcón C.R., Goodarzi H., Lee H., Liu X., Tavazoie S., Tavazoie S.F. (2015). HNRNPA2B1 is a mediator of m(6)A-dependent nuclear RNA processing Events. Cell.

[43] Ke S., Alemu E.A., Mertens C., Gantman E.C., Fak J.J., Mele A., Haripal B., Zucker-Scharff I., Moore M.J., Park C.Y., Vågbø C.B., Kusśnierczyk A., Klungland A., Darnell J.E., Darnell R.B. (2015). A majority of m6A residues are in the last exons, allowing the potential for 3’ UTR regulation. Genes Dev..

[44] Cooper G.M., Stone E.A., Asimenos G., NISC C.S.P., Green E.D., Batzoglou S., Sidow A. (2005). Distribution and intensity of constraint in mammalian genomic sequence. Genome Res..

[45] Davydov E.V., Goode D.L., Sirota M., Cooper G.M., Sidow A., Batzoglou S. (2010). Identifying a high fraction of the human genome to be under selective constraint using GERP++. Plos Comput. Biol..

[46] Bertolin A.P., Katz M.J., Yano M., Pozzi B., Acevedo J.M., Blanco-Obregón D., Gándara L., Sorianello E., Kanda H., Okano H., Srebrow A., Wappner P. (2016). Musashi mediates translational repression of the Drosophila hypoxia inducible factor. Nucleic Acids Res..

[47] Yano M., Okano H.J., Okano H. (2005). Involvement of Hu and heterogeneous nuclear ribonucleoprotein K in neuronal differentiation through p21 mRNA post-transcriptional regulation. J. Biol. Chem..

