# Supplementary material for: DGCR8-dependent efficient pri-miRNA processing of human pri-miR-9-2
Source: J Biol Chem. 2021 Feb 10;296:100409. doi: 10.1016/j.jbc.2021.100409 (PMC7995608; doi:10.1016/j.jbc.2021.100409)
Supplement: Figures S1–S4 [file mmc1.pdf]

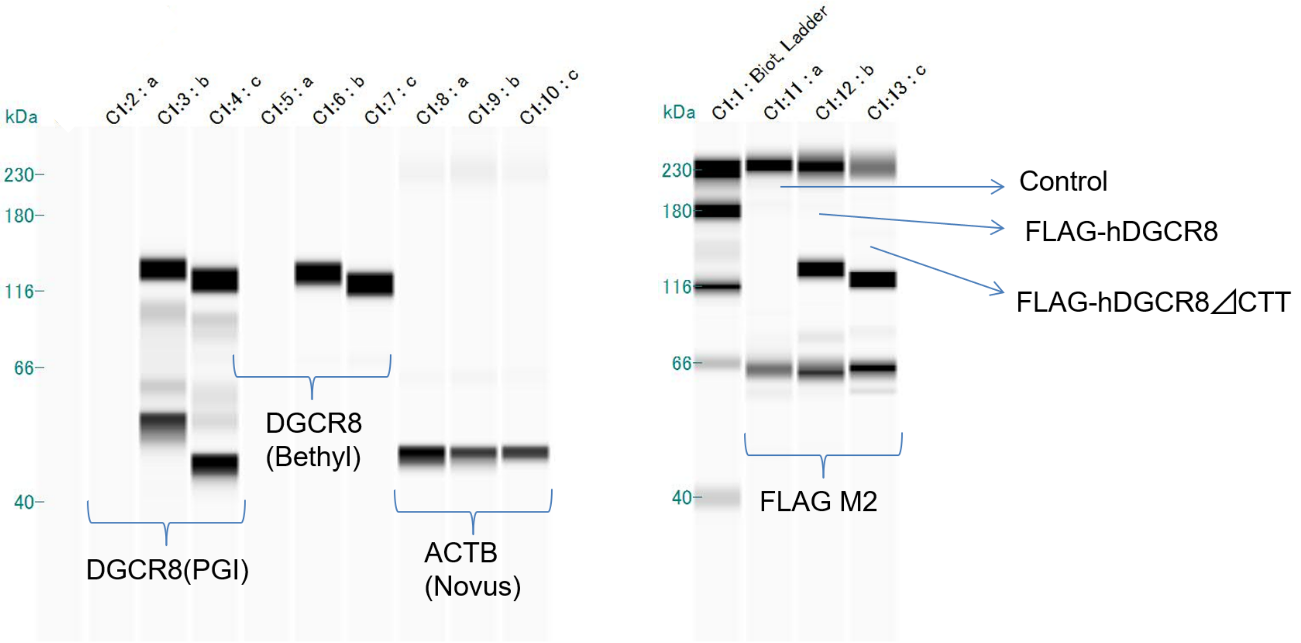

Supplemental Figure1. Overexpression of DGCR8

Control, FLAG-DGCR8 and FLAG-DGCR8 ΔCTT expression vectors were transfected into HeLa Tet-On 3G cells. DGCR8 and β-actin protein expression was analyzed with each antibodies by the Wes protein analysis system.

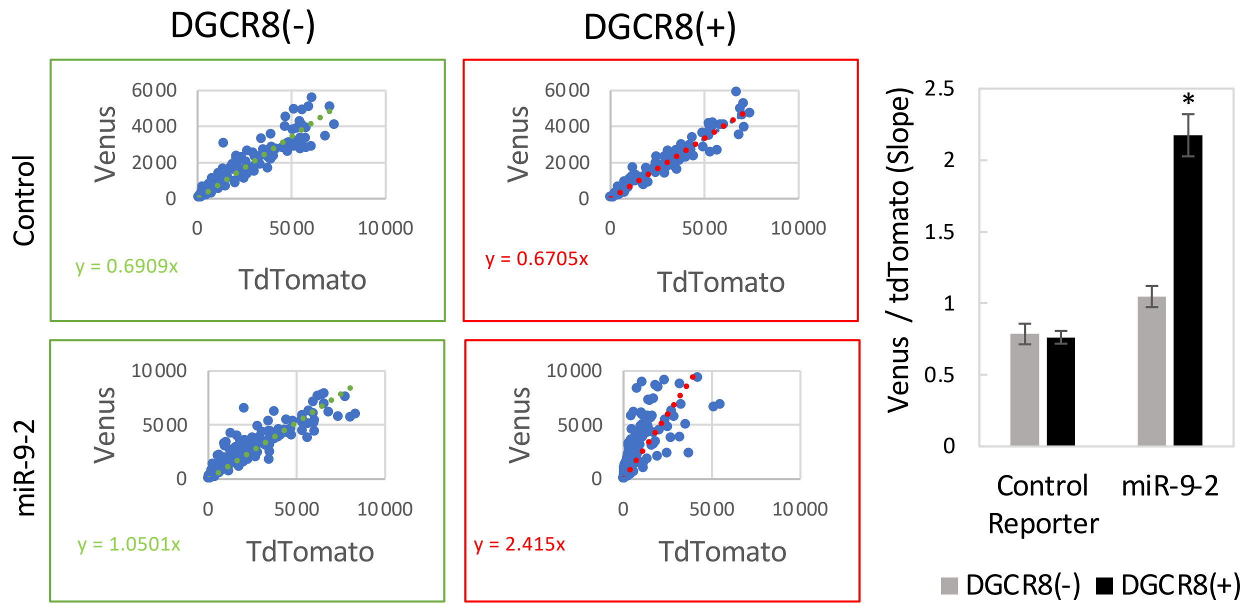

Supplemental Figure2. pri-miR-9-2 in DGCR8 dependent microprocessor activity A, pri-miRNA processing reporters containing Control, pri-miR-9-2 (300 nt) were subjected to the processing assay with HeLa Tet-On 3G cells. Graph indicates plots for each cell obtained from Venus and tdTomato fluorescence signals. Slope from linear regression are shown in each graph. Bar graph indicates slopes with or without DGCR8. Asterisk indicates significant change (t-test p<0.001)

pre-miR-9-2

GACAGAGGCCTGTGTGGGAAGCGAGTTGTTATCTTTGGTTATCT
AGCTGTATGAGTGTATTGGTCTTCATAAAGCTAGATAACCGAAA
GTAAAAACTCCTTCAAGATCGCCGGGGAGCGTG

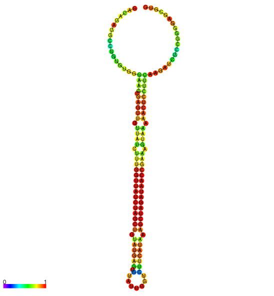

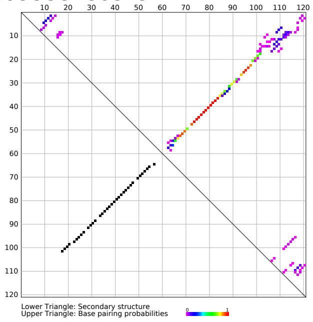

DRE

CGGGGAGCGTGTGAGAATGAAAGACTACAGCCGAGAGACAGTA
AAAACCAGAAAGGTCAGGAATACTTATTGAATCTAACTTTGTTTTT
GTTTTGTTTTT

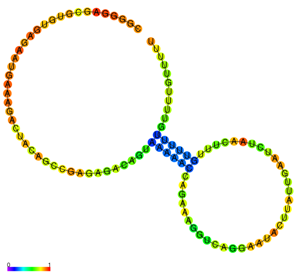

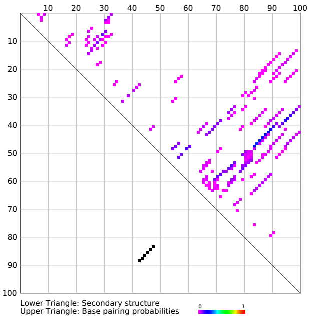

pre-miR-9-2 +DRE

GAGGCCTGTGTGGGAAGCGAGTTGTTATCTTTGGTTATCTAGC
TGTATGAGTGTATTGGTCTTCATAAAGCTAGATAACCGAAAGT
AAAAACTCCTTCAAGATCGCCGGGGAGCGTGTGAGAATGAAA
GACTACAGCCGAGAGACAGTAAAAACCAGAAAGGTCAGGAAT
ACTTATTGAATCTAACTTTGTTTTTGTTTTGTTTTT

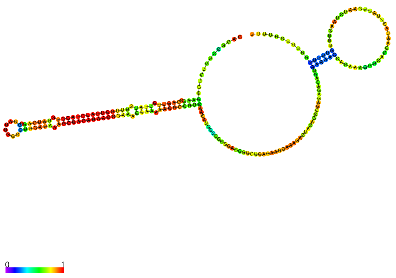

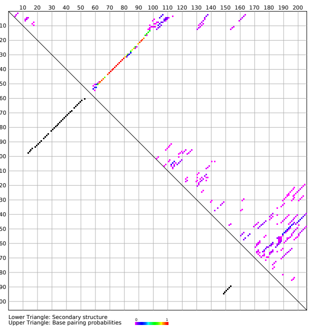

CentroidFold http://rtools.cbrc.jp

Supplemental Figure 3. Prediction of secondary structure for miR-9-2 DRE.

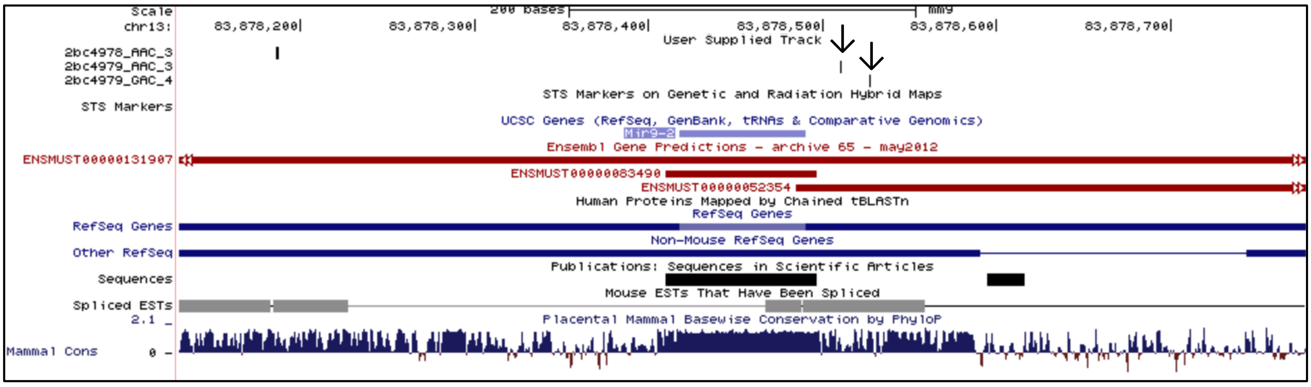

Supplemental Figure 4. m6A HITS-CLIP from mouse brain on pri-miR-9-2

Arrow indicates m6A sites around pri-miR-9-2
